# The association between METS-IR, an indirect index for insulin resistance, and lung cancer risk

**DOI:** 10.1093/eurpub/ckad234

**Published:** 2024-01-31

**Authors:** Guoqing Wang, Zhaopeng Zhu, Yi Wang, Qiang Zhang, Yungang Sun, Guanlian Pang, Wenjing Ge, Zhimin Ma, Huimin Ma, Linnan Gong, Hongxia Ma, Feng Shao, Meng Zhu

**Affiliations:** Department of Epidemiology, Center for Global Health, School of Public Health, Nanjing Medical University, Nanjing, China; Department of Epidemiology, Center for Global Health, School of Public Health, Nanjing Medical University, Nanjing, China; Department of Respiratory Disease, Nanjing Chest Hospital, Nanjing Medical University, Nanjing, China; Department of Thoracic Surgery, Nanjing Chest Hospital, Nanjing Medical University, Nanjing, China; Department of Thoracic Surgery, Nanjing Chest Hospital, Nanjing Medical University, Nanjing, China; Department of Epidemiology, Center for Global Health, School of Public Health, Nanjing Medical University, Nanjing, China; Department of Epidemiology, Center for Global Health, School of Public Health, Nanjing Medical University, Nanjing, China; Department of Epidemiology, Center for Global Health, School of Public Health, Nanjing Medical University, Nanjing, China; Department of Epidemiology, Center for Global Health, School of Public Health, Nanjing Medical University, Nanjing, China; Department of Epidemiology, Center for Global Health, School of Public Health, Nanjing Medical University, Nanjing, China; Department of Epidemiology, Center for Global Health, School of Public Health, Nanjing Medical University, Nanjing, China; Department of Thoracic Surgery, Nanjing Chest Hospital, Nanjing Medical University, Nanjing, China; Department of Epidemiology, Center for Global Health, School of Public Health, Nanjing Medical University, Nanjing, China; Department of Epidemiology, Jiangsu Key Lab of Cancer Biomarkers, Prevention and Treatment, Collaborative Innovation Center for Cancer Personalized Medicine, Nanjing Medical University, Nanjing, China

## Abstract

**Background:**

Insulin resistance has been reported to increase the risk of breast, prostate and colorectal cancer. However, the role of insulin resistance and its interaction with genetic risk in the development of lung cancer remains controversial. Therefore, we aimed to explore the association between a novel metabolic score for insulin resistance (METS-IR) and lung cancer risk.

**Methods:**

A total of 395 304 participants without previous cancer at baseline were included. The Cox proportional hazards regression model was performed to investigate the association between METS-IR and lung cancer risk. In addition, a Mendelian randomization analysis was also performed to explore the causal relationship. The joint effects and additive interactions between METS-IR and polygenetic risk score (PRS) of lung cancer were also investigated.

**Results:**

During a median follow-up of 11.03 years (Inter-quartile range (IQR): 10.30–11.73), a total of 3161 incident lung cancer cases were diagnosed in 395 304 participants. There was a significant association between METS-IR and lung cancer risk, with an HR of 1.28 (95% CI: 1.17–1.41). Based on the Mendelian randomization analysis, however, no causal associations were observed. We observed a joint effect but no interaction between METS-IR and genetic risk. The lung cancer incidence was estimated to be 100.42 (95% CI: 91.45–109.38) per 100 000 person-year for participants with a high METS-IR and PRS, while only 42.76 (95% CI: 36.94–48.59) with low METS-IR and PRS.

**Conclusions:**

High METS-IR was significantly associated with an increased risk of lung cancer. Keeping a low level of METS-IR might help reduce the long-term incident risk of lung cancer.

## Introduction

Lung cancer is second most common cancer, with an estimated 2.2 million new cases in 2020 worldwide.^1^ Although tobacco smoking is the most important risk factor for lung cancer, the identification of additional risk factors is also important for lung cancer prevention. Insulin resistance is a condition in which target tissues cannot respond to normal levels of insulin, which is often accompanied by the condition of obesity, metabolic syndrome, non-alcoholic fatty liver disease and other metabolic diseases.^2–4^ Evidence from large cohort studies also indicated that insulin resistance was a risk factor for overall cancer mortality.^5^ In addition, several studies also reported significant associations between insulin resistance and increased risk of breast, prostate and colorectal cancers.^6^ Even though some studies also linked insulin resistance with increased risk of lung cancer, the associations were inconsistent among different studies because of the small sample size or inconsistency of insulin resistance indicators.^7^^,^^8^

Evaluations of insulin resistance are often based on invasive methods, which limits its application in large cohort studies. Recently, a novel metabolic score for insulin resistance (METS-IR), which was based on glucose (mg/dl), triglycerides (mg/dl) and high-density lipoprotein cholesterol (HDL-C, mg/dl) along with body mass index (BMI), has shown a high concordance with the hyperinsulinemic-euglycemic clamp technique.^3^ Lung cancer is a polygenetic disease and several polygenic risk scores (PRSs) have been developed to quantify the genetic risk of lung cancer in recent studies.^9–12^ However, there is no study to explore the joint effect and interaction between METS-IR and PRS in the development of lung cancer.

Therefore, this study aimed to investigate the role of METS-IR in the development of lung cancer based on the UK Biobank by: (i) testing the association between METS-IR and lung cancer; (ⅱ) exploring the causal relationship by performing a genome-wide association study (GWAS) of METS-IR and followed by a Mendelian randomization (MR) analysis; and (ⅲ) estimating the joint effect and interaction between METS-IR and genetic risk in the development of lung cancer.

## Methods

### Study population

All data used in this study were derived from the UK Biobank. The detailed information about the UK Biobank is available online (http://www.ukbiobank.ac.uk/-wpcontent/uploads/2011/11/UK-Biobank-Protocol.pdf). In brief, more than 500 000 participants aged 40–69 years were recruited between 2006 and 2010 from 22 assessment centers in England, Wales and Scotland. All participants completed a self-administered touch-screen questionnaire, were given a brief computer-assisted interview and underwent physical measures. Meanwhile, biological samples were collected for various types of assays. All participants in the UK Biobank have provided a written informed consent form, and the UK Biobank has approval from the North West Multi-Center Research Ethics Committee as a Research Tissue Bank approval.

In this study, we excluded participants without the information of BMI, glucose, triglyceride and HDL-C (which were used to construct METS-IR), individuals with cancer at baseline and individuals who withdrew consent at the time of analysis. As a result, 395 304 eligible participants were included in the association study between METS-IR and lung cancer risk. On that basis, we further excluded participants without genetic information, with inferred sex mismatches or non-Caucasians, leaving 394 920 eligible participants for the GWAS and PRS analysis.

### METS-IR calculation

According to the study of Bello-Chavolla et al.,^3^ the METS-IR was calculated according to the following formula:

$$\mathrm{METS}-\mathrm{IR}=\frac{\ln\left[2\times \mathrm{Glucose}\;+\;\mathrm{Triglycerides}\right]\times \mathrm{BMI}}{\ln\left(\mathrm{HDL}-C\right)}.$$

The glucose was measured in mg/dl, the triglycerides were measured in mg/dl, the HDL-C was measured in mg/dl and the BMI was measured in kg/m^2^. In the UK Biobank, glucose was measured by hexokinase analysis on a Beckman Coulter AU5800, triglyceride was measured by GPO-POD analysis on a Beckman Coulter AU5800, HDL-C was measured by enzyme immunoinhibition analysis on a Beckman Coulter AU5800 and the BMI was calculated from measured height and weight [body mass (kg)/height (m^2^)].

### UKB genetic data

Detailed information about genotyping, imputation and quality control procedure of the UK Biobank was available in the UKB website (https://www.ukbiobank.ac.uk/-enable-your-research/about-our-data/genetic-data).^13^ Briefly, genome-wide genotyping was performed using the Affymetrix UK BiLEVE Axiom array or the Affymetrix UK Biobank Axiom array, which shared 95% of the markers. SHAPEIT3 and IMPUTE3 were used to impute ungenotyped single nucleotide polymorphisms (SNPs) based on the Haplotype Reference Consortium and 1000 Genomes Project. In this study, only SNPs with minor allele frequency >0.01 and Info score >0.8 were kept for further analysis.

### GWAS of METS-IR and MR analysis

To identify genetic variants associated with METS-IR, we performed a GWAS of METS-IR with BOLT-lmm software (v.2.3.4). Linear mixed model was used for the association analysis. Additional covariates included in BOLT-lmm included the genotyping array, the first 10 genetic principal components (PCs), age, sex, ethnicity, education, Townsend deprivation index, smoking, drinking and diagnosed diabetes.

To explore the causal relationship between METS-IR and lung cancer risk, two-sample MR analysis was conducted using the identified GWAS significant index SNPs of METS-IR and summary statistics of lung cancer from the International Lung Cancer Consortium. In addition to the inverse variance weighted (IVW) MR, MR Egger and weighted median MR were also performed to detect potential pleiotropic effects and the inclusion of invalid genetic instruments.

The MR analysis is based on three core assumptions: (i) the selected SNPs should be significantly associated with the METS-IR; (ii) the SNPs are not directly associated with lung cancer risk, and can only be causally associated with the METS-IR; and (iii) the SNPs must be independent of potential confounding factors between METS-IR and lung cancer. Therefore, we calculated *R*^2^ to represent the proportion of variance in the exposure explained by the SNPs, and also calculated the *F*-statistic to evaluate the strength of the SNPs, where *F* < 10 indicates weak strength of the SNPs (Supplementary methods); moreover, we also used MR-Egger regression intercept and its 95% confidence interval (95% CI) to test horizontal pleiotropy; at last, we adjusted for main confounding factors, such as age, gender, tobacco exposure and ethnic when calculating the GWAS summary statistics for METS-IR and lung cancer. All the MR analyses were performed using the TwoSampleMR (v.0.5.6) R package.^14–16^

In this study, the sample size of lung cancer GWAS is 85 716, with 29 266 cases and 56 450 controls, according to an online tool at http://cnsgenomics.com/shiny/mRnd/. We have 100% power to detect a potential causal association if the odds ratio (OR) is larger than 1.10 at a significance level of 0.05, assuming the SNPs explain a total of 23.31% variance of METS-IR.

### Lung cancer PRS calculation

To quantify the genetic risk of lung cancer, we constructed a PRS based on 18 SNPs, which were derived from the largest available published GWAS of lung cancer in the European population.^10^ All the SNPs needed for our analysis were available in the imputed database of UK Biobank. The PRS was calculated using the following formula:

$$\mathrm{PRS}=\sum_{j=1}^{M}\beta_{j}\times {\mathrm{SNP}}_{j}.$$

Of which, the SNPs were recorded as 0, 1 or 2 according to the number of risk alleles, and *β_j_* is the log OR for SNP_*j*_, which was obtained from the published GWAS of lung cancer. The participants were further categorized as low (lowest tertile), intermediate (middle tertile) and high (highest tertile) genetic risk according to the distribution of the PRS.^17^

### Outcome

In the UK Biobank, lung cancer events were collected by linking to the National Health Service central cancer and death registries in England, Wales and Scotland. Cancer outcomes were defined based on the 10th Revision of the International Classification of Diseases, of which lung cancer was defined as C33 and C34. Participants were followed up from the enrollment until the time of lung cancer diagnosis or censoring. In this study, censoring was defined as the time of death, withdrawal from the study or the complete date of follow-up (31 January 2021, for Scotland; and 29 February 2020, for England and Wales), whichever came first.

### Covariates

Sociodemographic and behavioral risk factors were included as covariates referring to previous studies.^7^ Briefly, age, sex, BMI, ethnicity, education level, Townsend deprivation index, smoking status, pack-years of smoking, alcohol intake frequency and diagnosed diabetes at baseline were included as covariates. These covariates were collected at baseline using a touch-screen questionnaire or measured by trained staff. Age was calculated according to the date of baseline assessment and birth date. Ethnic was defined as ‘White’ or ‘Non-white’. Education was defined as ‘Degree level or professional education’ or ‘Other levels’. Participants were divided into ‘Never smokers’ and ‘Former or current smokers’. The pack-years of smoking are calculated according to the number of cigarettes smoked per day and the number of years of smoking. Alcohol intake frequency was classified into six subgroups, including ‘Never’, ‘Special occasions only’, ‘One to three times a month’, ‘Once or twice a week’, ‘Three or four times a week’ and ‘Daily or almost daily’.

### Statistical analyses

Cox proportional hazard regression models were used to explore the association between METS-IR and lung cancer risk. Schoenfeld residuals were performed to examine the proportional hazards assumption. The hazard ratio (HR) and 95% CI were calculated for the scale per unit and 1-SD increase of METS-IR. Restricted cubic spline analysis was used to explore the possibly non-linear association shapes between METS-IR and lung cancer risk. Participants were also divided into three groups according to the tertiles of METS-IR and further classified as low (lowest tertile), intermediate (middle tertile) and high (highest tertile) METS-IR levels. As for missing covariates, multiple imputation was performed to impute the missing covariate data. Furthermore, subgroup analyses were also performed according to age, sex, BMI, smoking status and lung cancer histology.

To further explore the associations of METS-IR among different genetic risk groups, we performed joint effect and interaction analyses by further adjusting for the top 10 genetic PCs and genotyping batches in addition to the covariates mentioned above. The additive interactions were measured by the relative excess risk due to interaction (RERI) and the attributable proportion because of the interaction (AP).^12^ To estimate the 95% CI of RERI and AP, 5000 bootstraps were performed based on the estimation dataset.

To test the robustness of our findings, several sensitivity analyses were performed in our study: (i) we excluded participants without complete covariates; (ii) we excluded patients with lung cancer who were diagnosed during the first year of follow-up; and (iii) we restricted our study to individuals without diabetes at baseline. All analyses were performed with R (version 4.2.0), and the two-sided *P*-values of <0.05 were considered statistically significant in this study.

## Results

### Baseline characteristics

During a median follow-up of 11.03 years (IQR: 10.30–11.73), a total of 3161 incident lung cancer cases were diagnosed in 395 304 participants. Detailed information on incident lung cancer cases and other participants is shown in table 1. In summary, incident lung cancer cases tended to be older, less educated, males, smoking more tobacco, drinking more frequently and having higher levels of triglyceride, glucose and METS-IR.

**Table 1:** Distribution of population characteristics in individuals with and without lung cancer

Characteristics	Lung cancer (*N* = 3161)	No lung cancer (*N* = 392 143)
Age, year	61.69 (5.76)	56.24 (8.10)
Sex, *n* (%)		
Male	1653 (52.29)	181 859 (46.38)
Female	1508 (47.71)	210 284 (53.62)
Ethnic, *n* (%)		
White	3097 (97.98)	378 854 (96.61)
Non-White	64 (2.02)	13 289 (3.39)
Townsend index	−0.13 (3.56)	−1.31 (3.08)
Missing, *n* (%)	1 (0.03)	486 (0.12)
Education, *n* (%)		
Degree level/professional education	907 (28.69)	184 961 (47.17)
Other levels	2199 (69.57)	202 566 (51.66)
Missing	55 (1.74)	4616 (1.17)
Smoking status, *n* (%)		
Never	431 (13.64)	215 375 (54.92)
Past or current	2697 (85.32)	174 838 (44.59)
Missing	33 (1.04)	1930 (0.49)
Pack-year of smoking	32.73 (25.54)	8.06 (15.45)
Missing, *n* (%)	365 (11.55)	59 919 (15.28)
Alcohol intake frequency, *n* (%)		
Never	778 (24.61)	79 569 (20.29)
Special occasions only	584 (18.48)	91 047 (23.22)
One to three times a month	714 (22.59)	101 299 (25.83)
Once or twice a week	294 (9.30)	43 788 (11.17)
Three or four times a week	434 (13.73)	44 565 (11.36)
Daily or almost daily	348 (11.01)	31 023 (7.91)
Missing	9 (0.28)	852 (0.22)
BMI, kg/m^2^	27.43 (4.72)	27.44 (4.79)
HDL-C, mg/dl	53.45 (14.86)	55.96 (14.74)
Triglyceride, mg/dl	167.55 (91.10)	154.35 (90.89)
Glucose, mg/dl	94.17 (25.46)	92.11 (22.21)
METS-IR	41.09 (9.38)	40.27 (9.40)

Note: BMI, body mass index; METS-IR, metabolic score for insulin resistance.

### Association between METS-IR and lung cancer risk

**Figure 1:**
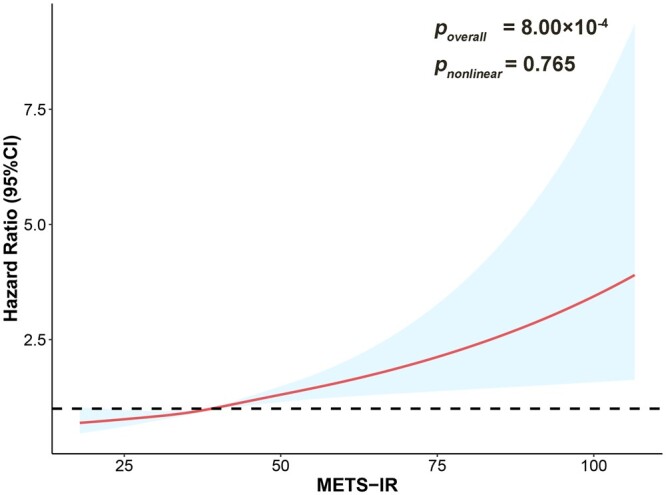
Association between the metabolic score for insulin resistance (METS-IR) and lung cancer risk based on restricted cubic spline (RCS) model in participants of UK Biobank. The RCS function with four knots for METS-IR, adjusted for age, sex, body mass index, Townsend index, education level, smoking status, alcohol intake frequency, pack-years of smoking, ethnicity and diabetes, was performed. Curves show hazard ratios (HRs) compared with the chosen reference METS-IR of 38.94.

Using unrestricted cubic spline analyses, we found METS-IR was significantly associated with increased lung cancer risk in a linear dependence (*P*_overall_ = 8.00 × 10^−4^ and *P*_nonlinear_ = 0.756; figure 1). There is a significant association between METS-IR and lung cancer (HR = 1.03, 95% CI: 1.02–1.04), and per SD increase of METS-IR was associated with an HR of 1.28 (95% CI: 1.17–1.41; table 2). Compared with participants at low METS-IR (the lowest tertile), participants at intermediate (the middle tertile) and high METS-IR (the top tertile) had a significantly higher risk of lung cancer, with HRs of 1.20 (95% CI: 1.08–1.33) and 1.34 (95% CI: 1.16–1.55), respectively (table 2).

**Table 2:** Associations of METS-IR with the risk of incident lung cancer in UK Biobank cohort

Index		No. cases/person-years	HR (95% CI)^a^	*P*-value^a^
Continued		3161/4 311 495	1.03 (1.02, 1.04)	2.79 × 10^−7^
Per SD		3161/4 311 495	1.28 (1.17, 1.41)	2.79 × 10^−7^
Category	Low	888/1 443 285	1.00 (ref)	
	Intermediate	1098/1 438 142	1.20 (1.08, 1.33)	5.94 × 10^−4^
	High	1175/1 430 068	1.34 (1.16, 1.55)	9.89 × 10^−5^

Note: HR, hazards ratio; 95% CI, 95% confidence interval; METS-IR, metabolic score for insulin resistance; Low, the lowest tertile of METS-IR; Intermediate, the middle tertile of METS-IR; High, the top tertile of METS-IR.

a Adjusted for age, sex, BMI, Townsend index, education level, smoking status, alcohol intake frequency, pack-years of smoking, ethnicity and diabetes.

Stratified analyses showed that the association effects of METS-IR on the risk of lung cancer were more significant in females (*P*_heterogeneity_ = 0.005) and those with BMI < 30 kg/m^2^ (*P*_heterogeneity_ = 0.003), while no heterogeneity was observed among other subgroups (Supplementary table S1). In the sensitivity analyses, similar associations were observed after exclusion of participants without complete covariates, diagnosed with lung cancer during the first year of follow-up, or with diabetes at baseline (Supplementary tables S2–S4).

### Genetic loci associated with METS-IR and MR analysis with lung cancer

In the UK Biobank, 66 805 SNPs were associated with METS-IR (*P *<* *5 × 10^−8^; Supplementary figure S1). After removing SNPs that were in linkage disequilibrium (*r*^2^ < 0.1), 1277 SNPs were remaining for use in the MR analyses, of which 819 SNPs were available in the lung cancer GWAS. The 819 SNPs was strongly associated with METS-IR with the mean *F*-statistic of 146 and explained ∼23.31% of the variance of METS-IR in this study.

In the IVW MR analysis, we observed no causal association between METS-IR and lung cancer risk (OR = 1.00, 95% CI: 0.99–1.01; Supplementary figure S2). No significant association was identified in other MR models. Supplementary figure S3 shows scatter plots of the associations between METS-IR and lung cancer. Funnel plots are provided in Supplementary figure S4.

### Joint effect and interaction between METS-IR and genetic risk on lung cancer

**Figure 2:**
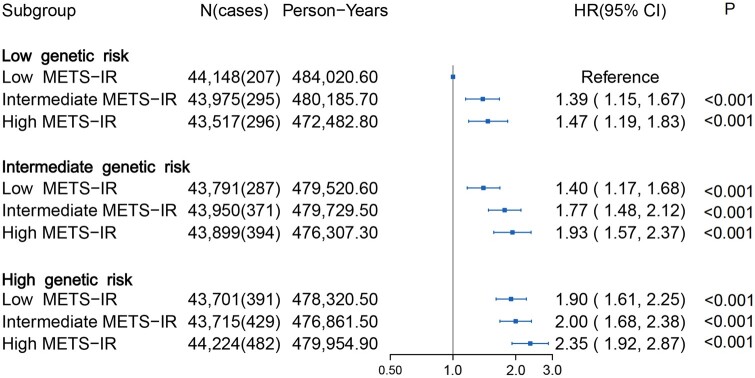
Risk of incident lung cancer according to metabolic score for insulin resistance (METS-IR) and genetic categories. The hazard ratios (HRs) were estimated using Cox proportional hazard models with adjustment age, sex, body mass index, Townsend index, education level, smoking status, alcohol intake frequency, pack-years of smoking, ethnicity, diabetes and the first 10 principal components of ancestry.

The PRS of lung cancer was significantly associated with increased lung cancer risk in a linear and dose–response manner (Supplementary figure S5). We also observed a joint effect of METS-IR and PRS on the risk of lung cancer; i.e. the overall risk of lung cancer increased with both METS-IR and PRS (figure 2). Of participants with a high METS-IR and PRS (the top tertile), the incidence rates of lung cancer were estimated to be 100.42 (95% CI: 91.45–109.38) per 100 000 person-year compared with 42.76 (95% CI: 36.94–48.59) per 100 000 person-year in participants with low METS-IR and PRS (the lowest tertile), with an HR of 2.35 (95% CI: 1.92–2.87) (Supplementary table S5 and figure 2). After stratifying participants by genetic risk, we found that there was a significant reduction in the 5-year absolute risk of incident lung cancer in participants with low METS-IR across different genetic risk groups (Supplementary figure S6). However, in the additive interaction analysis, the 95% CIs of the RERI and AP included 0, which indicated there were no significant interactions between METS-IR and genetic risk (Supplementary table S6).

## Discussion

In this study, based on a large-scale prospective cohort, we observed that METS-IR, a novel indicator of IR, was significantly associated with an increased risk of lung cancer. Further analysis showed a joint effect between METS-IR and PRS, and nearly 16% of lung cancer could be attributed to abnormal METS-IR. However, MR analysis demonstrated no causal associations between genetically predicted METS-IR and lung cancer risk. These results collectively demonstrated that the METS-IR was probably a biomarker for lung cancer, while not directly driving the carcinogenesis of lung cancer.

IR is characterized by decreased insulin function despite an increased amount of insulin.^18^ In addition, it is reported that IR is related to dyslipidemia, hypertension and type 2 diabetes.^19^^,^^20^ There are many methods to measure IR, which are mainly divided into direct or indirect measurement. Direct measurement refers to the glucose clamp technique,^21^ but it is invasive and complex. Meanwhile, indirect measurement also can be divided into two groups, including insulin-based and non-insulin-based indices. The insulin-based measures of IR included the homeostatic model assessment for insulin resistance (HOMA-IR) and the quantitative insulin sensitivity check index,^22^ while the non-insulin-based measures included the triglycerides and glucose index (TyG index), the combination index of triglycerides, glucose and BMI (TyG–BMI index), the ratio of triglycerides and HDL-c (TG/HDL-c ratio) and the METS-IR.^23–25^ Among these indexes, METS-IR plays a good diagnostic performance for IR and is suitable for application in large-scale epidemiological studies because of its simple composition.^26^

Several studies have investigated the associations between IR biomarkers and lung cancer risk. Based on 81 lung cancer cases and 162 controls, Petridou et al.^8^ have reported a marginally significant association between HOMA-IR and lung cancer risk in Greece (OR = 2.02, 95% CI: 0.88–4.65). Argirion et al.^7^ also validated the positive association between HOMA-IR and lung cancer risk (HR = 1.83, 95% CI: 0.99–3.38) based on the Alpha-Tocopherol, Beta-Carotene Cancer Prevention Study of Finnish men. Although similar effects were observed, no significant association was found because of the small sample size in the two studies. Recently, Yan et al.^27^ reported that another biomarker of IR, the TyG index, was associated with the development of lung cancer (OR = 3.651, 95% CI: 2.461–5.417) in a case–control study; however, the study by Wang et al.,^28^ based on 324 000 participants from the UK Biobank, showed no association between TyG index and lung cancer risk (HR = 0.91, 95% CI: 0.64–1.18). By then, there was no study to investigate the association between METS-IR and lung cancer risk. To our knowledge, this study is the first to reveal that higher METS-IR was positively associated with an increased risk of lung cancer.

Several potential mechanisms have been proposed to explain the positive association between IR and cancer. Firstly, the high levels of insulin in IR individuals may promote cancer via abnormal stimulation of multiple cellular signaling cascades, i.e. the Ras signal pathway, enhancing growth factor-dependent cell proliferation or directly affecting cell metabolism.^29^ Secondly, the high levels of insulin increase the bioavailability and expression of insulin-like growth factor binding proteins 1 and 2,^30^ and these IGFs act jointly with tobacco carcinogens to enhance lung carcinogenesis.^31^ Moreover, it has been reported that IR was usually accompanied by a low-grade inflammatory state characterized by the overproduction of proinflammatory cytokines such as interleukin-6 and tumor necrosis factor-alpha. These cytokines can further increase the cancer risk via the nuclear factor kappa-B pathway.^32^ However, the underlying molecular mechanism concerning the association observed in this study remains to be determined.

We used two-sample MR analyses to evaluate the causal association between METS-IR and lung cancer. Overall, we did not find a causal association between METS-IR and lung cancer risk, which was not consistent with the findings of observational analysis. The disagreement between observational and MR analyses was also observed in several previous studies.^33–36^ Although MR analysis can reduce the bias caused by unmeasured confounders and give causal estimates, the low magnitude of genetically determined exposure usually leads to a null association in MR analysis.^37^ In this study, potential confounding factors have been fully adjusted and several sensitivity analyses have shown similar association effects, which demonstrate that the conflict was not likely to be biased by confounding or reverse causality.^38^ Therefore, the conflict between MR and observational analysis indicates that the METS-IR may not be the cause of lung cancer while being a biomarker of lifestyles (i.e. smoking, diet, physical exercise and body status). Although it is not feasible to reduce the risk of lung cancer incidence through targeted intervention of METS-IR, monitoring the levels of METS-IR could predict the changes in lung cancer risk in a timely manner. Moreover, we found that keeping a low METS-IR was significantly associated with a reduced risk of lung cancer across different genetic risk groups, which indicated that the indicator is applicable to the whole population.

There are some advantages in our study. Firstly, the large sample size and prospective design of the UK Biobank could increase the credibility of our results. Secondly, we applied GWAS and MR analysis for the first time to investigate genetic variation associated with METS-IR and the causal relationship between METS-IR and the risk of lung cancer. Finally, to test the stability and reliability of our results, we performed several stratified analyses and sensitivity analyses. However, we acknowledged that there are still some limitations to this study. Firstly, participants were mostly White, which may limit our ability to extrapolate results to other races. Secondly, there is only baseline information but no follow-up information in this study. Finally, missing covariates were imputed using multiple imputations, which may result in bias in our results, although similar findings were observed before and after the exclusion of participants with incomplete covariates.

## Conclusion

In summary, we observed a significant association between high METS-IR and increased risk of lung cancer based on the UK Biobank. However, we found no causal association between METS-IR and lung cancer (LC) in the MR analysis. These results demonstrated that METS-IR was likely to be a biomarker for lung cancer risk without directly driving lung cancer carcinogenesis.

## Supplementary Material

ckad234_Supplementary_Data

## Data Availability

The datasets generated and/or analyzed during this study are available from the corresponding author upon reasonable request.
