# Supplementary material for: The association between METS-IR, an indirect index for insulin resistance, and lung cancer risk
Source: Eur J Public Health. 2024 Jan 31;34(4):800–5. doi: 10.1093/eurpub/ckad234 (PMC11293818; doi:10.1093/eurpub/ckad234)
Supplement: ckad234_Supplementary_Data [file ckad234_supplementary_data.zip › ckad234_Supplementary_Data/ejph-2023-08-om-0465-File004.docx]

**Supplementary appendix**

**Supplementary Figure 1** Manhattan plot of a GWAS of METS - IR among 394,920 participants in UK Biobank

**Supplementary Figure 2** Association of METS-IR and lung cancer in MR analyses

**Supplementary Figure 3** Scatter plot of Mendelian randomized analysis of METS - IR and lung cancer

**Supplementary Figure 4** Funnel plot of Mendelian randomized analysis of METS-IR and LC

**Supplementary Figure 5** Association of the decile of PRS with incident LC

**Supplementary Figure 6** Absolute risk over 5 years of lung cancer according to PRS and METS-IR joint distribution

**Supplementary Table 1** Stratified analyses for the association between METS-IR and risk of LC based on complete information of each stratified factors

**Supplementary Table 2** Sensitivity analyses restricted to participants with complete covariates

**Supplementary Table 3** Sensitivity analyses after excluding incident cases during the first year of follow-up

**Supplementary Table 4** Sensitivity analyses after excluding participants with diabetes based on complete information of diabetes

**Supplementary Table 5** Incidence of LC per 100,000 person-year according to PRS and METS-IR

**Supplementary Table 6** RERI and AP for Additive Interaction between METS-IR and Genetic Categories

**Supplementary Figure 1** Manhattan plot of a GWAS of METS - IR among 394,920 participants in UK Biobank

**
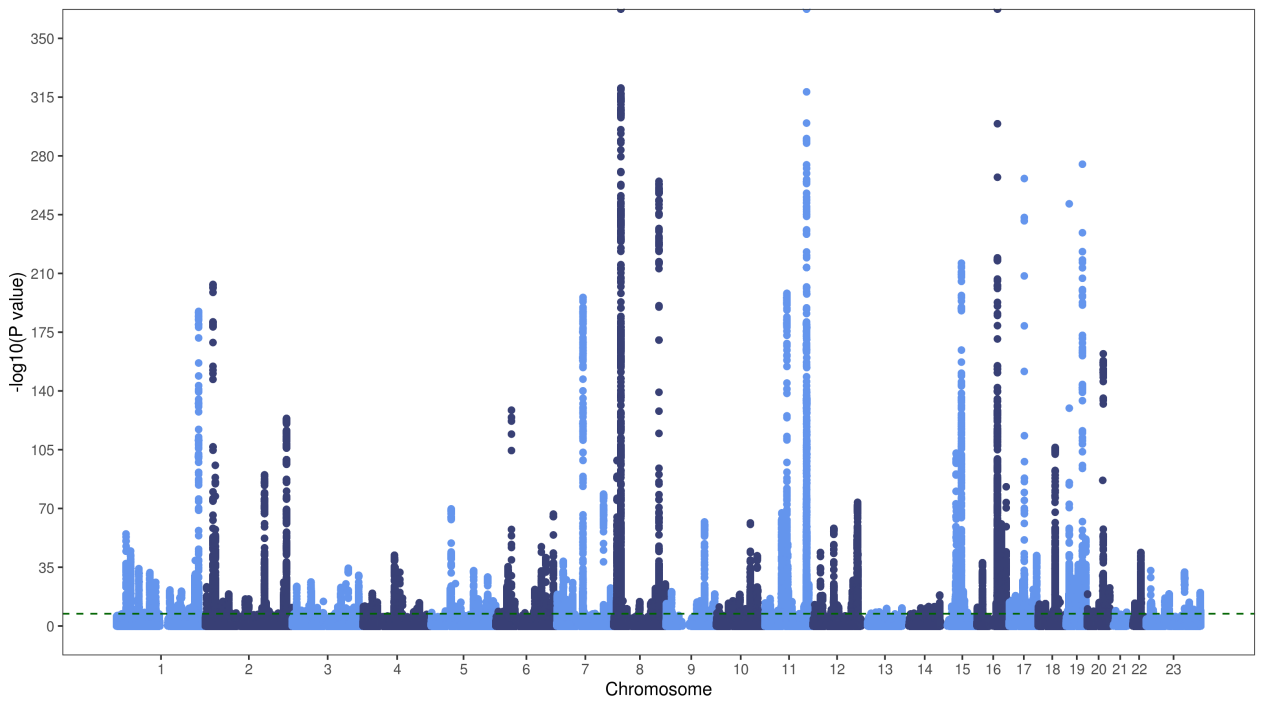
**

The association of SNPs with METS-IR across chromosomes. The -log_10_(P value) from the linear mixed model using the BOLT-lmm, adjusted for genotyping array, the first 10 genetic principal components (PCs), age, sex, ethnicity, education, Townsend deprivation index, smoking, drinking, and diagnosed diabetes, were plotted across chromosomes. Bonferroni-corrected significance levels (5×10^-8^) are indicated by dotted line in green.

**Supplementary Figure 2** Association of METS-IR and lung cancer in MR analyses

**
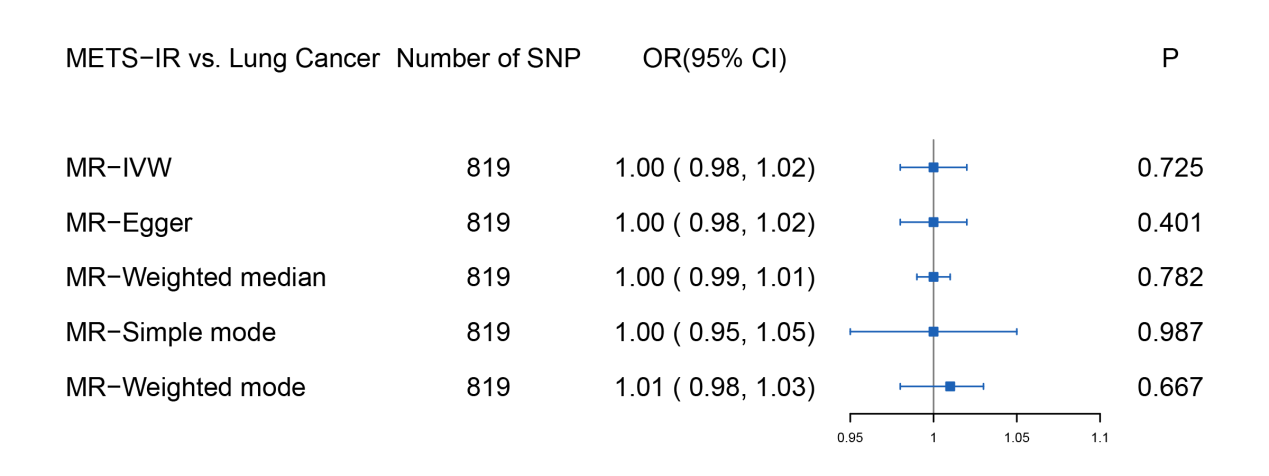
**

Results are shown for the different methods of Mendelian randomization (MR) analyses used in this study: inverse variance weighted (IVW), MR-Egger, weighted median, weighted mode and simple mode. Abbreviations: IVW, inverse variance weighted; MR, mendelian randomization; SNPs, single‐nucleotide polymorphisms; OR, odds ratio.

**Supplementary Figure 3** Scatter plot of Mendelian randomized analysis of METS - IR and lung cancer


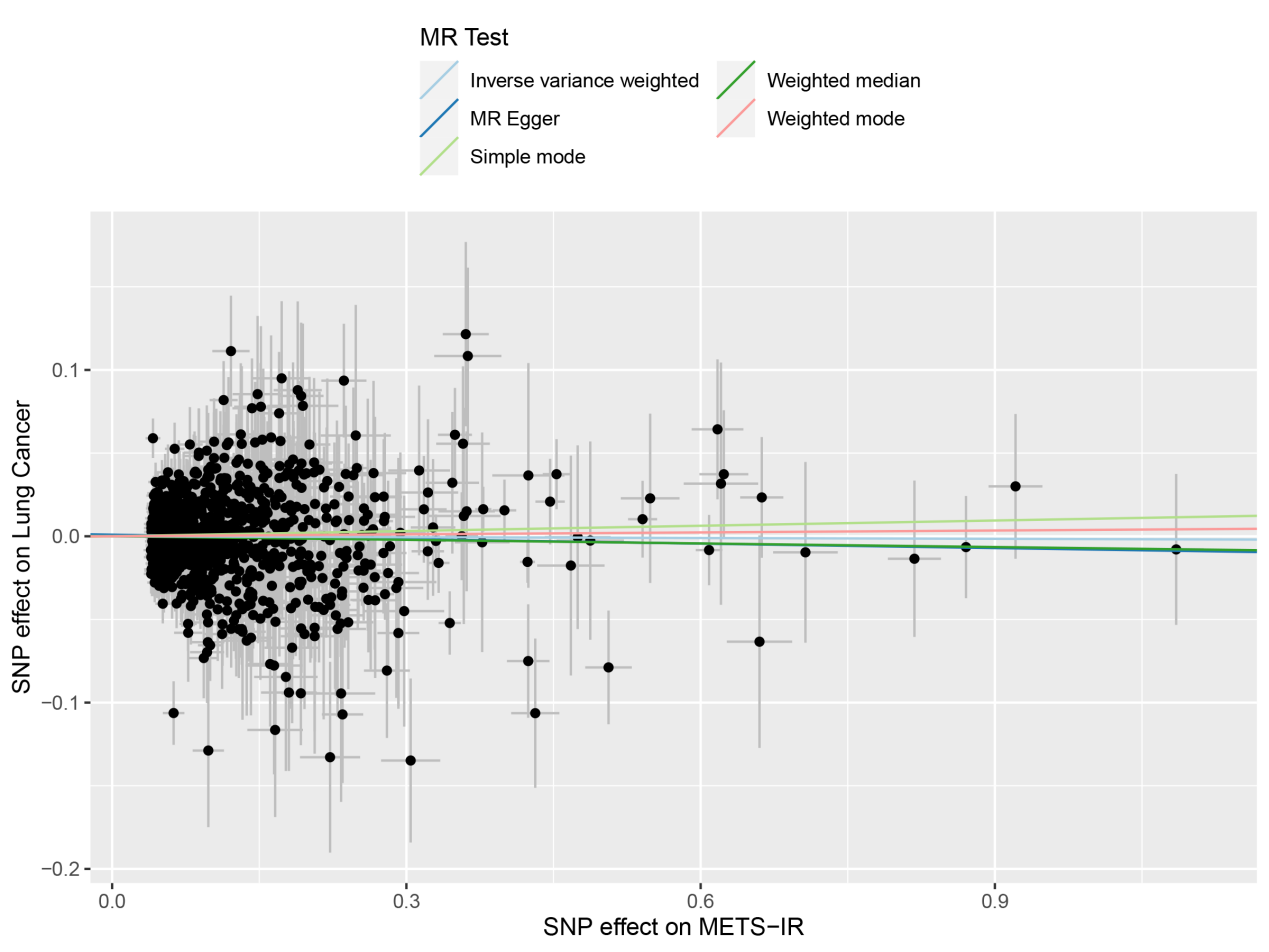


Scatter plot of the association between METS-IR and lung cancer. The five methods applied in the current manuscript were all depicted. Lines in light blue, blue, light green, green and red represent IVW, MR-Egger, simple mode, weighted median, and weighted mode methods.

**Supplementary Figure 4** Funnel plot of Mendelian randomized analysis of METS-IR and LC


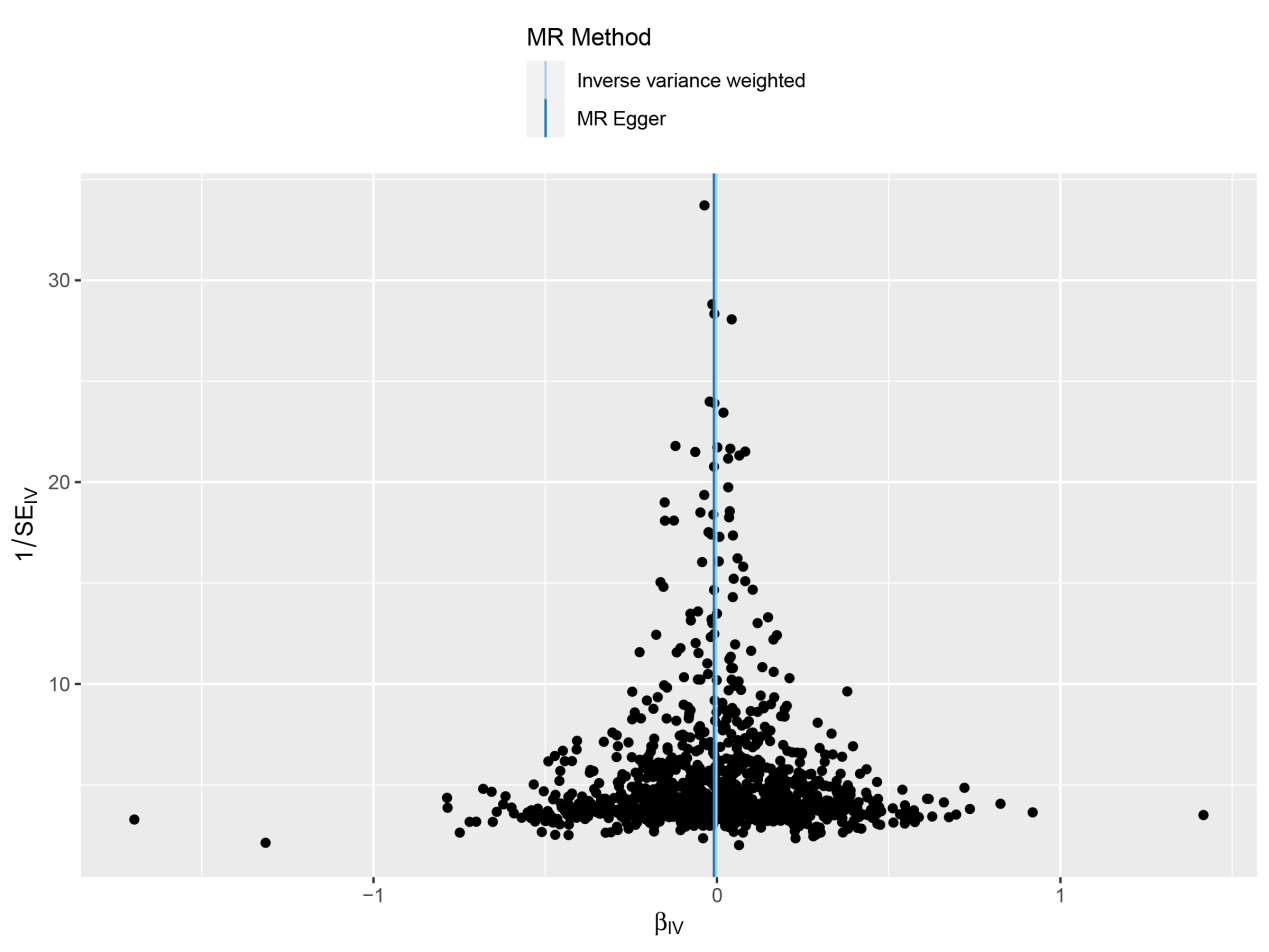


Funnel plot showing the inverse variance weighted MR estimate of each METS-IR SNP with lung cancer versus 1/standard error (1/SEIV).

**Supplementary Figure 5** Association of PRS with incident LC


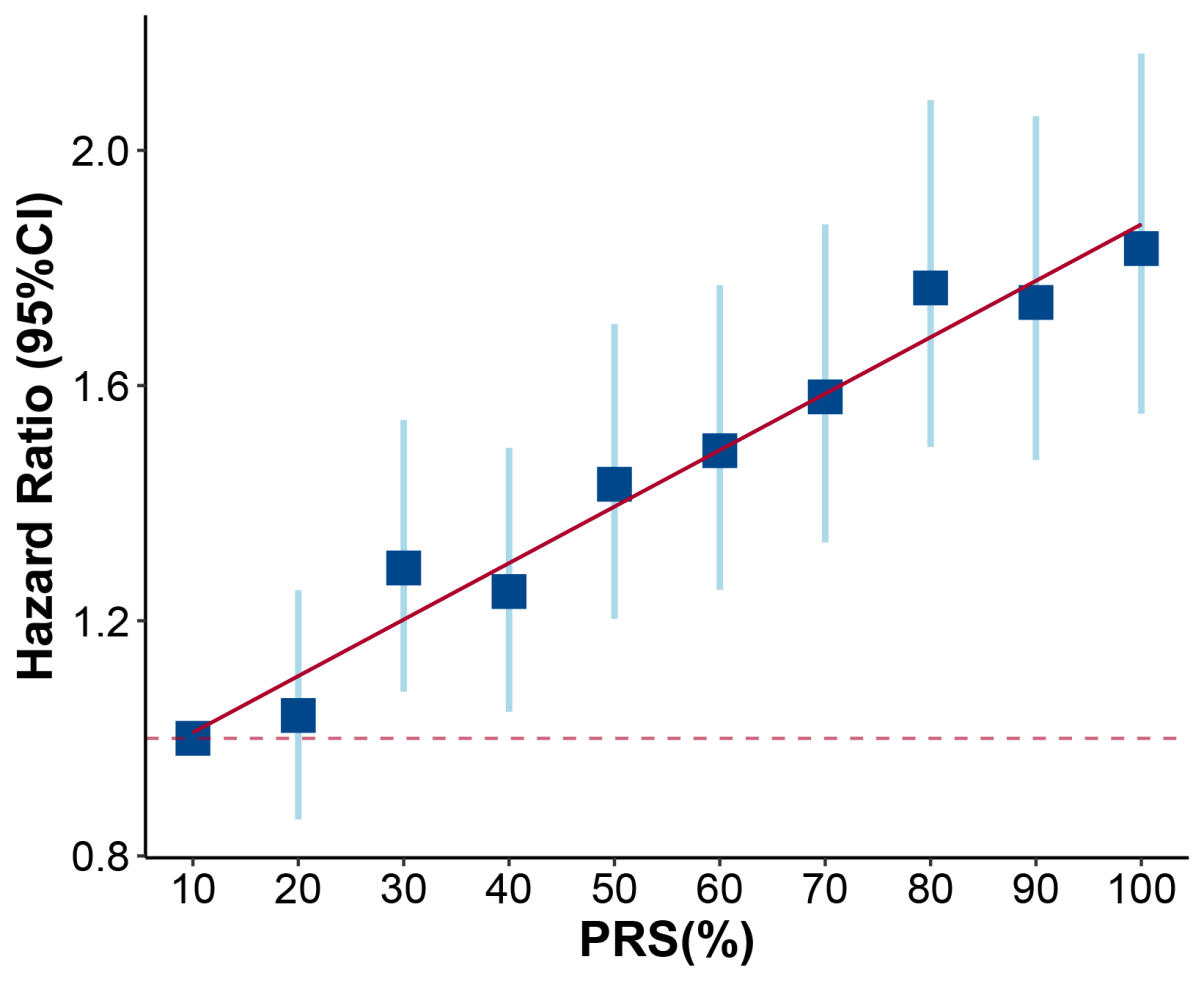


Participants were divided into ten equal groups according to the PRS, and the HRs of each group were compared with those in the bottom decile of the PRS. Error bars are 95% CIs. HRs and 95% CIs were estimated using Cox proportional-hazard models with adjustment for age, sex, BMI, Townsend index, education level, smoking status, alcohol intake frequency, pack-years of smoking, ethnicity, diabetes, the top 10 principal components of ancestry, and genotyping batch.

Definition of abbreviations: PRS, polygenic risk score; HR, hazards ratio; CI, confidence interval.

**Supplementary Figure 6** Absolute risk over 5 years of lung cancer according to PRS and METS-IR joint distribution


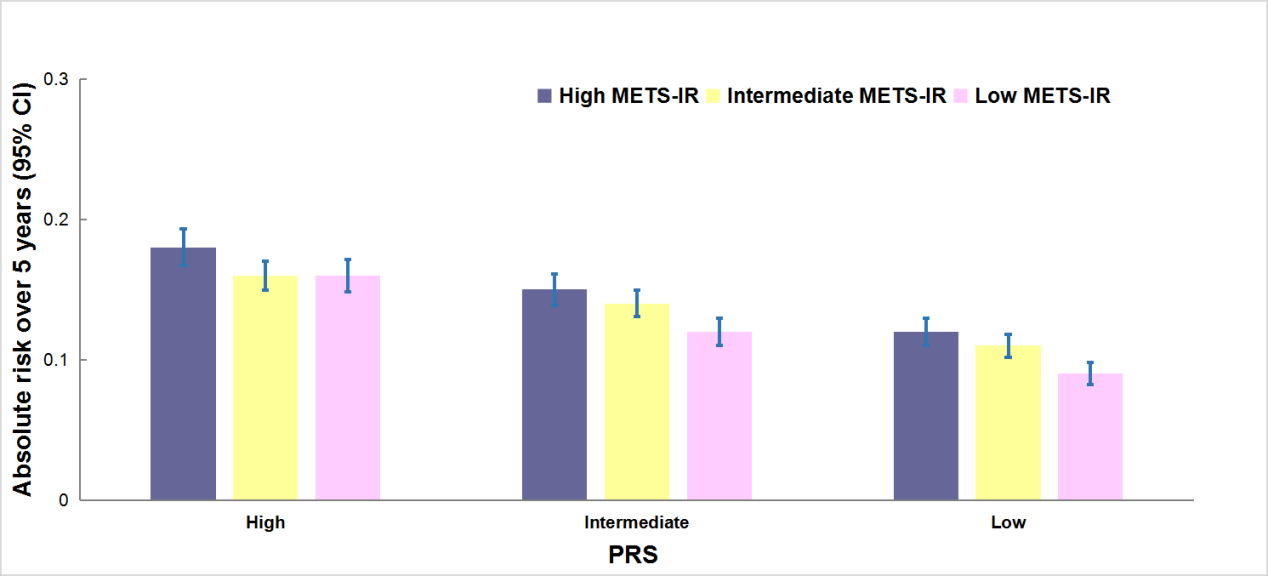


The absolute risk over 5years of incident lung cancer associated with METS-IR (Low, the lowest tertile of METS-IR; Intermediate, the middle tertile of METS-IR; High, the top tertile of METS-IR), was assessed and stratified by genetic risk (Low, the lowest tertile of PRS; Intermediate, the middle tertile of PRS; High, the top tertile of PRS). Adjusted for age, sex, BMI, Townsend index, education level, smoking status, alcohol intake frequency, pack-years of smoking, ethnicity, and diabetes.

**Supplementary Table 1** Stratified analyses for the association between METS-IR and risk of LC based on complete information of each stratified factors

| **Group** | **METS-IR^*^** | | |
| --- | --- | --- | --- |
|  | **HR (95%CI)** | **P value** | **P heterogeneity** |
| Sex |  |  | 0.005 |
| Male | 1.02 (1.00, 1.03) | 0.020 |  |
| Female | 1.05 (1.03, 1.06) | 1.36×10-8 |  |
| Age |  |  | 0.158 |
| <60 (year) | 1.04 (1.02, 1.06) | 7.17×10-5 |  |
| >=60 (year) | 1.02 (1.01, 1.03) | 3.91×10-4 |  |
| BMI |  |  | 0.003 |
| <30 (kg/m2) | 1.04 (1.03, 1.05) | 5.80×10-9 |  |
| >=30 (kg/m2) | 1.01 (0.99, 1.03) | 0.250 |  |
| Smoking status |  |  | 0.612 |
| Never smoker | 1.03 (1.00, 1.06) | 0.067 |  |
| Current or past smoker | 1.04 (1.03, 1.05) | 2.39×10-10 |  |
| Histology |  |  |  |
| AD | 1.02 (1.00, 1.03) | 0.032 | 0.139 |
| SC | 1.04 (1.02, 1.07) | 7.24×10-5 |  |
| SLC | 1.04 (1.00, 1.07) | 0.026 |  |

Definition of abbreviations: HR, hazards ratio; CI, confidence interval; METS-IR, the metabolic score for insulin resistance.

^*^ Adjust for age, BMI, Townsend index, education level, smoking status, alcohol intake frequency, pack-years of smoking, ethnicity, and diabetes when available.

**Supplementary Table 2** Sensitivity analyses restricted to participants with complete covariates

| **Index** | | **No. cases /**  **person-years** | **HR (95%CI) ^*^** | P value | P trend |
| --- | --- | --- | --- | --- | --- |
|  |  |  |  |  |  |
| **METS-IR** |  |  |  |  |  |
| **Continued** |  | 2,738/3,602,515 | 1.02 (1.01, 1.03) | 5.50×10-5 |  |
| **Per SD** |  | 2,738/3,602,515 | 1.23 (1.11, 1.36) | 5.50×10-5 |  |
| **Category** | Low | 791/1,205,587 | 1.00 (ref) |  | 0.008 |
|  | Intermediate | 933/1,201,791 | 1.12 (1.00, 1.25) | 0.045 |  |
|  | High | 1,014/1,195,137 | 1.24 (1.06, 1.45) | 0.008 |  |

Definition of abbreviations: HR, hazards ratio; CI, confidence interval; METS-IR, the metabolic score for insulin resistance; Low, the lowest tertile of METS-IR; Intermediate, the middle tertile of METS-IR; High, the top tertile of METS-IR.

**^*^** Adjust for age, sex, BMI, Townsend index, education level, smoking status, alcohol intake frequency, pack-years of smoking, ethnicity, and diabetes.

**Supplementary Table 3** Sensitivity analyses after excluding incident cases during the first year of follow-up

| **Index** | | **No. cases /**  **person-years** | **HR (95%CI) ^*^** | P value | P trend |
| --- | --- | --- | --- | --- | --- |
|  |  |  |  |  |  |
| **METS-IR** |  |  |  |  |  |
| **Continued** |  | 2,989/4,311,399 | 1.03 (1.02, 1.04) | 8.82×10-7 |  |
| **Per SD** |  | 2,989/4,311,399 | 1.28 (1.16, 1.41) | 8.82×10-7 |  |
| **Category** | Low | 844/1,443,111 | 1.00 (ref) |  | 3.94×10-4 |
|  | Intermediate | 1,031/1,438,207 | 1.17 (1.05, 1.30) | 0.003 |  |
|  | High | 1,114/1,430,081 | 1.31 (1.13, 1.53) | 4.21×10-4 |  |

Definition of abbreviations: HR, hazards ratio; CI, confidence interval; METS-IR, the metabolic score for insulin resistance; Low, the lowest tertile of METS-IR; Intermediate, the middle tertile of METS-IR; High, the top tertile of METS-IR.

**^*^** Adjust for age, sex, BMI, Townsend index, education level, smoking status, alcohol intake frequency, pack-years of smoking, ethnicity, and diabetes.

**Supplementary Table 4** Sensitivity analyses after excluding participants with diabetes based on complete information of diabetes

| **Index** | | **No. cases /**  **person-years** | **HR (95%CI) ^*^** | P value | P trend |
| --- | --- | --- | --- | --- | --- |
|  |  |  |  |  |  |
| **METS-IR** |  |  |  |  |  |
| **Continued** |  | 2,869/4,080,950 | 1.03 (1.02, 1.04) | 9.52×10-9 |  |
| **Per SD** |  | 2,869/4,080,950 | 1.33 (1.21, 1.47) | 9.52×10-9 |  |
| **Category** | Low | 824/1,363,995 | 1.00 (ref) |  | 2.81×10-4 |
|  | Intermediate | 1,002/1,360,020 | 1.18 (1.06, 1.32) | 0.002 |  |
|  | High | 1,043/1,356,936 | 1.33 (1.14, 1.55) | 2.99×10-4 |  |

Definition of abbreviations: HR, hazards ratio; CI, confidence interval; METS-IR, the metabolic score for insulin resistance; Low, the lowest tertile of METS-IR; Intermediate, the middle tertile of METS-IR; High, the top tertile of METS-IR.

**^*^**Adjust for age, sex, BMI, Townsend index, education level, smoking status, alcohol intake frequency, pack-years of smoking, and ethnicity.

**Supplementary Table 5** Incidence of LC per 100,000 person-year according to PRS and METS-IR

| **PRS^†^** | **METS-IR^*^** | **Number of LC** | **Person-years** | **Incidence (per 100,000 person-year)** | **Lower** | **Upper** |
| --- | --- | --- | --- | --- | --- | --- |
| Low | Low | 207 | 484,020.6 | 42.76 | 36.94 | 48.59 |
|  | Intermediate | 295 | 480,185.7 | 61.43 | 54.42 | 68.44 |
|  | High | 296 | 472,482.8 | 62.64 | 55.51 | 69.78 |
| Intermediate | Low | 287 | 479,520.6 | 59.85 | 52.92 | 66.77 |
|  | Intermediate | 371 | 479,729.5 | 77.33 | 69.46 | 85.20 |
|  | High | 394 | 476,307.3 | 82.71 | 74.55 | 90.88 |
| High | Low | 391 | 478,320.5 | 81.74 | 73.64 | 89.84 |
|  | Intermediate | 429 | 476,861.5 | 89.96 | 81.44 | 98.47 |
|  | High | 482 | 479,954.9 | 100.42 | 91.45 | 109.38 |

Definition of abbreviations: HR, hazards ratio; CI, confidence interval; METS-IR, the metabolic score for insulin resistance.

**^*^** The METS-IR was divided into low (the bottom tertile), intermediate (the second tertile), and high (the top tertile) risk groups.

**^†^** The genetic risk was divided into low (the bottom tertile), intermediate (the second tertile), and high (the top tertile) risk groups according to the distribution of PRS.

**Supplementary Table 6** RERI and AP for Additive Interaction between METS-IR and Genetic Categories

| **METS-IR ^*^** | **PRS ^†^** | | | |
| --- | --- | --- | --- | --- |
|  | Intermediate | | High | |
|  | RERI (95%CI)^‡^ | AP (95%CI) ^‡^ | RERI (95%CI) ^‡^ | AP (95%CI) ^‡^ |
| Intermediate | -0.02(-0.17, 0.13) | -0.01(-0.09, 0.07) | -0.29(-0.47, -0.13) | -0.15(-0.23, -0.06) |
| High | 0.05(-0.10, 0.20) | 0.03(-0.05, 0.10) | -0.03(-0.20, 0.14) | -0.01(-0.09, 0.06) |

Definition of abbreviations: RERI, relative excess risk due to interaction; AP, attributable proportion due to interaction; CI, confidence interval; METS-IR, the metabolic score for insulin resistance.

**^*^** The METS-IR was divided into low (the bottom tertile), intermediate (the second tertile), and high (the top tertile) risk groups.

**^†^** The genetic risk was divided into low (the bottom tertile), intermediate (the second tertile), and high (the top tertile) risk groups according to the distribution of PRS.

^‡^ To estimate the RERI and AP, the low METS-IR category and the lowest genetic risk (low PRS) groups were the reference categories. Adjust for age, sex, BMI, Townsend index, education level, smoking status, and alcohol intake frequency, pack-years of smoking, ethnicity, and diabetes.
