# Supplementary material for: The association between METS-IR, an indirect index for insulin resistance, and lung cancer risk
Source: Eur J Public Health. 2024 Jan 31;34(4):800–5. doi: 10.1093/eurpub/ckad234 (PMC11293818; doi:10.1093/eurpub/ckad234)
Supplement: ckad234_Supplementary_Data [file ckad234_supplementary_data.zip › ckad234_Supplementary_Data/ejph-2023-08-om-0465-File005.doc]

***Supplemental methods***

***Genetic instrument for METS-IR***

The R^2^ was calculated to represent the the proportion of variance in the exposure explained by the IVs:

Where *EAF* indexes the minor allele frequency, *beta* represents the effect size of SNP on exposure, *SE(beta)* is the standard error for beta, and *N* represents sample size of exposure.

The F statistic was calculated to test the statistical significance of the correlation between the instrument variables and CRP:

where *R^2^* represents the variability explained by instrumental variable (*R^2^*=0.2331), *N* indexes the number of individuals (n=394920), and *k* refers to the number of SNPs (*k*=819). Cumulatively, the *F*-statistic was 146, which indicates a strong relationship between instrumental variable and METS-IR.
